# Multiple chimeric antigen receptors successfully target chondroitin sulfate proteoglycan 4 in several different cancer histologies and cancer stem cells

**DOI:** 10.1186/2051-1426-2-25

**Published:** 2014-08-19

**Authors:** Rachel E Beard, Zhili Zheng, Kiran H Lagisetty, William R Burns, Eric Tran, Stephen M Hewitt, Daniel Abate-Daga, Shannon F Rosati, Howard A Fine, Soldano Ferrone, Steven A Rosenberg, Richard A Morgan

**Affiliations:** 1 Surgery Branch, Center for Cancer Research, National Cancer Institute, 10 Center Drive, Building 10 Hatfield CRC, Rm 3-5930, 20892-1201 Bethesda, MD, USA; 2 Laboratory of Pathology, Center for Cancer Research, National Cancer Institute, 10 Center Drive, Building 10 Hatfield CRC, MSC 4605, 20892-1201 Bethesda, MD, USA; 3 Division of Hematology and Medical Oncology, New York University Langone Medical Center, New York, New York, USA; 4 Department of Surgery, Massachusetts General Hospital, 55 Fruit Street, Boston, MA 02114, USA; 5 Current address: Bluebird bio, 150 Second St, Cambridge, MA 02141, USA

**Keywords:** Immunotherapy, *CSPG4*, Chimeric antigen receptor, Cancer stem cells, Melanoma, Glioblastoma

## Abstract

**Background:**

The development of immunotherapy has led to significant progress in the treatment of metastatic cancer, including the development of genetic engineering technologies that redirect lymphocytes to recognize and target a wide variety of tumor antigens. Chimeric antigen receptors (CARs) are hybrid proteins combining antibody recognition domains linked to T cell signaling elements. Clinical trials of CAR-transduced peripheral blood lymphocytes (PBL) have induced remission of both solid organ and hematologic malignancies. Chondroitin sulfate proteoglycan 4 (CSPG4) is a promising target antigen that is overexpressed in multiple cancer histologies including melanoma, triple-negative breast cancer, glioblastoma, mesothelioma and sarcoma.

**Methods:**

CSPG4 expression in cancer cell lines was assayed using flow cytometry (FACS) and reverse-transcription PCR (RT-PCR). Immunohistochemistry was utilized to assay resected melanomas and normal human tissues (n = 30) for CSPG4 expression and a reverse-phase protein array comprising 94 normal tissue samples was also interrogated for CSPG4 expression. CARs were successfully constructed from multiple murine antibodies (225.28S, TP41.2, 149.53) using second generation (CD28.CD3ζ) signaling domains. CAR sequences were cloned into a gamma-retroviral vector with subsequent successful production of retroviral supernatant and PBL transduction. CAR efficacy was assayed by cytokine release and cytolysis following coculture with target cell lines. Additionally, glioblastoma stem cells were generated from resected human tumors, and CSPG4 expression was determined by RT-PCR and FACS.

**Results:**

Immunohistochemistry demonstrated prominent CSPG4 expression in melanoma tumors, but failed to demonstrate expression in any of the 30 normal human tissues studied. Two of 94 normal tissue protein lysates were positive by protein array. CAR constructs demonstrated cytokine secretion and cytolytic function after co-culture with tumor cell lines from multiple different histologies, including melanoma, breast cancer, mesothelioma, glioblastoma and osteosarcoma. Furthermore, we report for the first time that CSPG4 is expressed on glioblastoma cancer stem cells (GSC) and demonstrate that anti-CSPG4 CAR-transduced T cells recognize and kill these GSC.

**Conclusions:**

The functionality of multiple different CARs, with the widespread expression of CSPG4 on multiple malignancies, suggests that CSPG4 may be an attractive candidate tumor antigen for CAR-based immunotherapies using appropriate technology to limit possible off-tumor toxicity.

## Background

The development of the field of immunotherapy has led to significant progress in the treatment of metastatic cancer. The most effective immunotherapy treatment option to date is the adoptive cell transfer (ACT) of autologous tumor-infiltrating lymphocytes (TIL) that can induce complete durable regression of metastatic melanomas [1]. This therapy necessitates the surgical resection of a tumor deposit and subsequent generation of TIL, which is not possible in every patient, and to date has shown limited applicability in histologies other than melanoma.

Genetic engineering technologies that allow for the redirection of lymphocytes to recognize and target a variety of tumor antigens has created new possibilities for the utilization of immunotherapy to treat metastatic cancer. T cell receptor (TCR) genes that target specific tumor antigens have been inserted into peripheral blood lymphocytes (PBL) and, when transfused back into patients in conjunction with administration of high-dose interleukin-2 after non-myeloablative chemotherapy, have been shown to effect tumor regression in patients with melanoma and synovial cell sarcoma [2-5]. Chimeric antigen receptors (CARs) have also been investigated as an alternative way to redirect T cells to recognize and destroy tumor cells. Comprised of a single-chain variable fragment (scFv) from a monoclonal antibody linked to intracellular T cell signaling domains, these fusion proteins are able to effect antigen recognition in a manner that is not restricted to the major histocompatibility complex, conferring a distinct advantage over TCRs [6-8]. CAR-transduced T cells have been shown to induce remission of both solid organ and hematologic malignancies [9-15].

We sought to develop an optimally effective CAR to target chondroitin sulfate proteoglycan 4 (CSPG4). Formerly known as High Molecular Weight-Melanoma Associated Antigen (HMW-MAA), this highly immunogenic cell surface proteoglycan was identified on melanoma cells in the 1970’s and has been shown to facilitate the progression from radial to vertical growth in melanoma tumors [16-18]. It has also shown overexpression and potential as an immunotherapy target on a number of other tumor histologies including triple-negative breast cancer, head and neck squamous cell cancer, mesothelioma and glioblastoma [16,19-21]. Our previous work demonstrated the ability to construct a CSPG4-specific CAR using the murine monoclonal antibody (mAb) 225.28S. PBL transduced with the 225.28S CAR demonstrated cytokine secretion and cytolytic function when co-cultured with melanoma cell lines *in vitro* and were reactive against explanted human melanomas [22]. Herein we expand upon that work by utilizing different murine mAbs reactive against CSPG4 to construct CARs that target cell lines from multiple tumor histologies as well as cancer stem cells (CSC).

## Results

### CSPG4 expression in tumor cell lines and normal tissues

**Figure 1:**
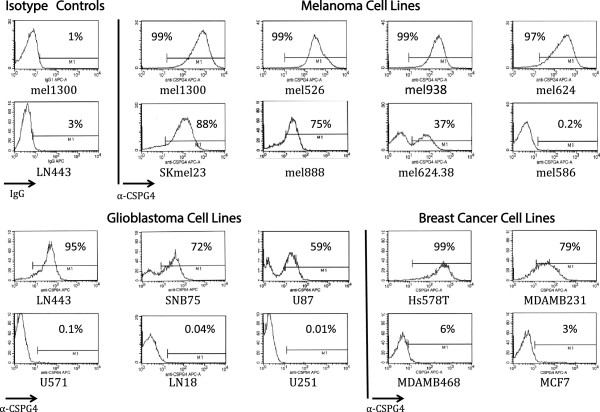
**CSPG4 expression in tumor cell lines from multiple histologies by FACS.** Fluorescence-activated cell sorting analysis (FACS) was performed using a conjugated mAb (anti-hNG2/MCSP) specific for human chondroitin sulfate proteoglycan 4 (CSPG4) according to manufacturers’ recommendations (R&D Systems, Minneapolis, MD). Representative isotype controls are shown in the top left hand panels. Cell lines and percent expression were as identified.

**Figure 2:**
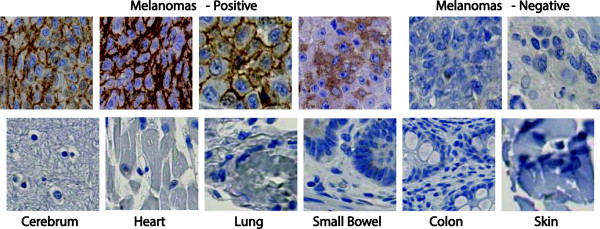
**Immunohistochemistry demonstrates staining of melanoma tumors and no staining of any normal tissues.** Staining was done with the TP41.2 antibody. 30 normal tissues [adrenal, bladder, bone marrow, breast (5 samples), cerebellum, cerebrum grey matter, cerebrum white matter, colon (2 samples), esophagus, heart, kidney cortex, kidney medulla, liver (2 samples), lung (2 samples), lymph node, mesothelium, muscle, ovary, pancreas, peripheral nerve, prostate, salivary gland, skin (2 samples), small bowel, spleen, stomach, testis, thyroid, uterus endometrium, uterus myometrium] were tested with representative data shown here. Magnification shown is 40X.

**Figure 3:**
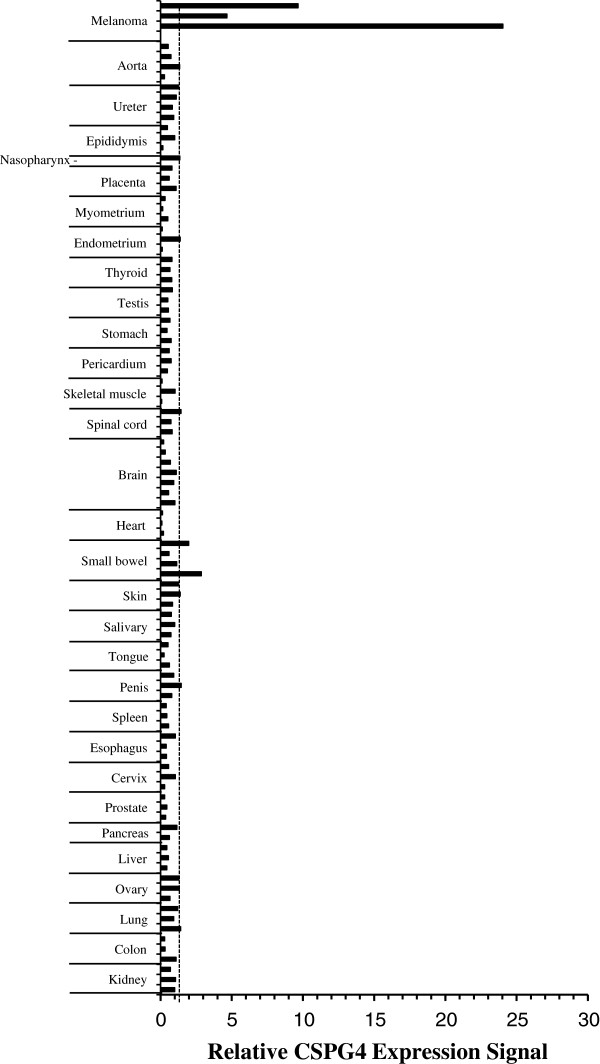
**Reverse-phase protein array.** Total proteins were extracted from frozen tissues and applied to Multi-Spot™ plates (see Methods). Anti-CSPG4 (TP41.2) and anti-Actin antibodies were applied and following incubation and wash, detected with SULFO-TAG™ antibodies. Signal was normalized to actin expression and is expressed as relatively fold over background (water). The horizontal dashed line is the threshold antigen detection value set as the background mean plus one standard deviation. Each bar is protein from a different donor from the indicated tissues.

Cell lines from multiple histologies were studied for CSPG4 expression by fluorescence-activated cell sorting analysis (FACS) (Figure 1). Six of the 8 melanoma lines were strongly positive for CSPG4 expression with an additional line, mel624.38, demonstrating intermediate expression. Of the 6 glioblastoma cell lines assayed, 3 demonstrated CSPG4 expression, as did 2 of the 4 triple-negative breast cancer cell lines. To further analyze CSPG4 expression in tumors and normal tissues, we used immunohistochemistry and protein array technology for antigen detection. Immunohistochemistry using antibody TP41.2 failed to demonstrate any significant staining on a normal tissue panel, with 30 normal tissue types tested, but showed antibody staining of melanoma samples in a membranous pattern (Figure 2). To further analyze CSPG4 antigen expression we used a reverse-phase protein array technology, which immobilizes protein lysates from frozen normal tissues on a carbon fiber surface. Antibody TP41.2 was again used for detection and after normalization for loading with beta-actin, the threshold level for antigen expression was set to the mean background level plus one standard deviation (value, 1.203). In this assay the relative CSPG4 antigen expression in three melanoma samples was 4.668, 9.665, and 24.041 (Figure 3). Of 94 normal tissues tested, we observed CSPG4 antigen detection above the threshold level in 2 of 4 small bowel samples (values, 1.982 and 2.875, Figure 3).

### CARs from murine antibodies recognize cell lines from multiple cancer histologies

**Figure 4:**
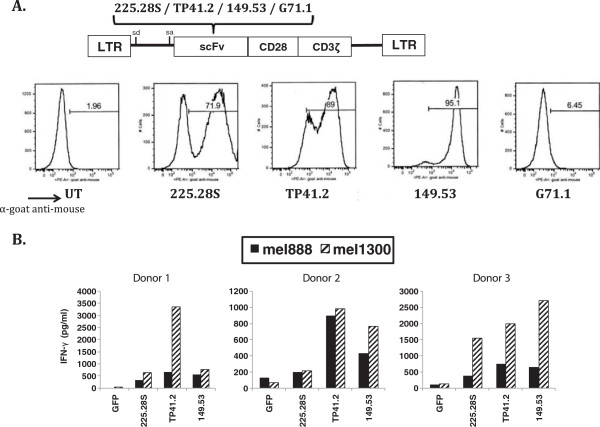
**Murine CARs targeting CSPG4.** Transduction efficiencies by FACS was determined using goat α-mouse Fab staining **(A)** for CAR detection. Data shown is representative of FACS data from three different PBL donors. Cells were cocultured with melanoma tumor cell lines to determine effector cytokine release. IFN-γ release when transduced PBL were co-cultured with CSPG4-positive melanoma target lines **(B)**. Mel888 and mel1300 = melanoma cell lines. Anti-CSPG4 CAR-transduced T cells activity was statistically significant (p < 0.05, student’s t-test) in all comparisons to GFP control transduced T cells with the exception of 225.28 s transduced T cells from donor 2 (p > 0.05).

**Figure 5:**
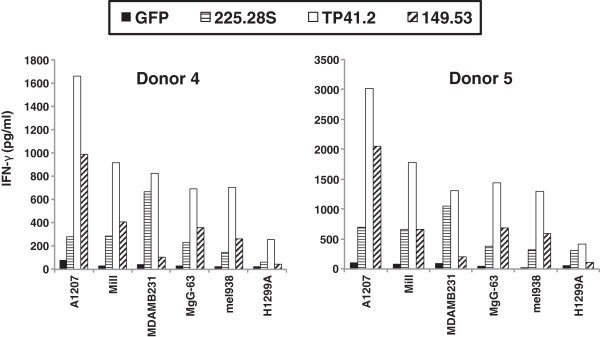
**PBL transduced with murine CARs targeting CSPG4 recognize tumor lines from multiple cancer histologies.** All CARs shown use the second generation CD28.CD3ζ signaling domain. A1207 = glioblastoma multiforme cell line, Mill = mesothelioma, MDAMB231 = breast cancer cell lines, MgG-63 = osteosarcoma cell line, mel938 = melanoma cell lines, H1299A = non-small cell lung cancer line (negative control). Anti-CSPG4 CAR-transduced T cells activity was statistically significant (p < 0.05, student’s t-test) in all comparisons to GFP control transduced T cells versus H1299 cells from donor 4. Anti-CSPG4 CAR-transduced T cells activity was statistically significant (p < 0.05) in all comparisons of TP41.2 and 149.53 CAR transduced cells to GFP control transduced T cells versus H1299 cells from donor 5. There was no significant difference in activity of 225.28S CAR transduced cells to GFP control transduced T cells versus H1299 cells from donor 5 in cocultures with MgG-63 and mel938 cells (p > 0.05).

**Figure 6:**
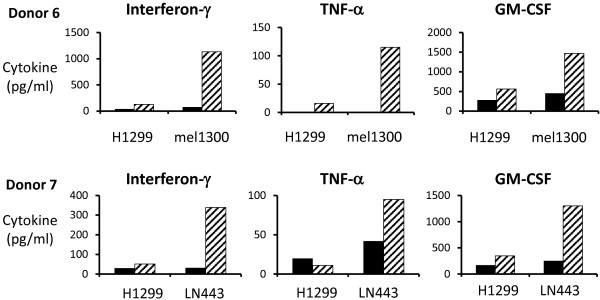
**CAR transduced T cell produce multiple effector cytokines.** TP41.2 anti-CSPG4 CAR transduced T cells from two different donors (donors 6 and 7) were cocultured with melanoma (mel1300) and glioblastoma (LN443) tumor cell lines to determine effector cytokine release (H1299 was used as a CSPG4 negative control line). IFN-γ, TNF-α, and GMCSF production was determined by ELISA (mean of triplicate determinations). Anti-CSPG4 CAR-transduced T cells activity (stripped bars) was statistically significant (p < 0.05, student’s t-test) in all comparisons to GFP control (black bars) transduced T cells.

CARs were constructed from four different murine scFv fragments: 225.28S, TP41.2, 149.53 and G71.1 all of which have demonstrated reactivity with CSPG4 [23-25]. These were cloned into a MSGV1-based retroviral vector with the CD28.CD3ζ signaling domains and then transduced into PBL from different donors. The CARs from the mAbs 225.28S, TP41.2 and 149.53 antibodies were detected on the surface of transduced PBL by FACS, whereas the CAR from the G71.1 antibody was not (Figure 4A). DNA sequence analysis of the G71.1 CAR vector did not reveal any cause for the lack of expression. The three CARs that demonstrated surface expression on PBL also demonstrated IFN-γ release when cocultured with the CSPG4-expressing melanoma lines mel1300 and mel888 (Figure 4B). Though cytokine release varied between PBL donors, cytokine release from the TP41.2-CD28.CD3ζ construct was consistently high in all three donors.PBL from two additional donors were transduced with the three reactive CARs and cocultured with CSPG4-positive cell lines from multiple different histologies including the glioblastoma cell line A1207, the breast cancer cell line MDAMB231, the mesothelioma cell line Mill, the osteosarcoma cell line MgG-63 and the melanoma line mel938. Each of the CSPG4 CAR transduced PBL cultures yielded IFN-γ effector cytokine release with considerably lower levels observed with the CSPG4-negative non-small cell lung cancer line H1299 (Figure 5). Again the CAR constructed from the TP41.2 antibody was the most reactive overall.We next determined the ability of the CSPG4 CAR transduced T cells to produce multiple cytokines (Figure 6). TP41.2-based CSPG4 CAR transduced T cells were cocultured with either melanoma line mel1300 or glioblastoma cell line LN443. Cocultures were performed with two different donor T cells and included the H1299 negative control cell line and GFP transduced T cells as a specificity control. Results presented in Figure 6 demonstrate that both donor T cells were capable of producing three effector cytokines; interferon-gamma (IFN-γ), tumor necrosis factors alpha (TNF-α), and granulocyte macrophage colony stimulating factor (GM-CSF). These effector cytokine production was specific to tumor lines expressing CSPG4, as minimal cytokine was release in coculture with H1299 cells or with GFP vector transduced cells.

### Glioblastoma stem cells express CSPG4 and are recognized by CSPG4-targeting CAR transduced cells

**Figure 7:**
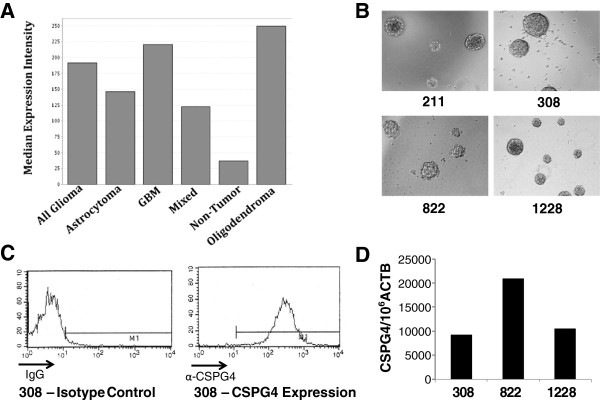
**CSPG4 has potential to treat brain malignancies. (A)** CSPG4 gene expression has been demonstrated in several different brain tumors by Affymetrix analysis. Data from the Rembrandt database. National Cancer Institute. 2005. REMBRANDT home page. http://rembrandt.nci.nih.gov. Accessed 2013 April 12. **(B)** Glioblastoma stem cells generated from all tumors grow as gliospheres and express CSPG4 by FACS **(C)** and by RT-PCR **(D)**. Representative data shown.

CSPG4 expression in glioblastoma cell lines motivated us to query a large database of gene expression in human brain tumor samples (Rembrandt database, National Cancer Institute, http://rembrandt.nci.nih.gov) [26]. Compared to adjacent non-tumor tissues, CSPG4 was overexpressed in all brain tumors samples (Figure 7A). Glioblastoma cell lines have been previously shown to exhibit gene expression patterns different from that of primary brain tumors. Alternatively, glioma stem cells (GSC) lines derived from primary tumors and grown as gliospheres have been shown to be much more representative of primary tumors [27]. Using multiple GSC lines (Figure 7B), we determined that these lines expressed CSPG4 by both FACS (Figure 7C) and RT-PCR based assays (Figure 7D).

**Figure 8:**
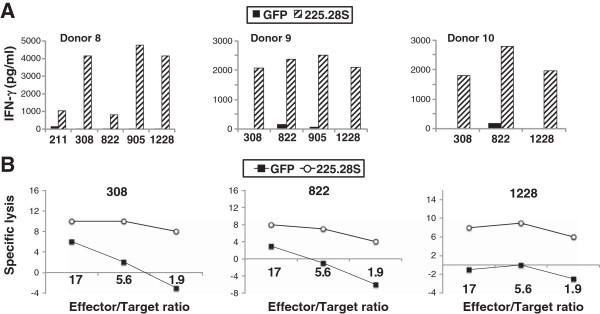
**CSPG4 CAR recognition of glioma stem cells. (A)** PBL from 3 donors were transduced with anti-CSPG4 murine CAR 225.28S-CD28.CD3ζ and co-cultured with the indicated GSC lines. Shown is the resultant IFN-γ release (pg/ml) following overnight co-culture (values are the average of duplicate determinations). **(B)** CSPG4 CAR-transduced PBL were mixed with the indicated GSC lines at various effector to target ratios and cytolytic function determined (shown as percent specific lysis). Data are representative of duplicate determinations.

We initiated coculture experiments to determine if CSPG4 CAR-engineered T cells recognized these GSC lines and observed that CSPG4 CAR transduced cells recognized all five GSC lines that were tested (Figure 8A). Although it was difficult to disaggregate and then label gliospheres with ^51^Cr, we also determined that three of three GSC lines tested could be specifically lysed by CSPG4 CAR transduced T cells (Figure 8B).

## Discussion

CSPG4 is an antigen that has been widely studied and has previously been suggested as a target for immunotherapy. It is expressed on a high percentage of melanomas, greater than 80% in some reports, and immunohistochemistry demonstrates expression in several other malignancies, including triple-negative breast cancer, glioblastoma, head and neck squamous cell carcinoma, sarcoma and mesothelioma [16,21,28,29]. Studies have demonstrated the effectiveness of targeting CSPG4 with monoclonal antibody (mAb)-based immunotherapy, both *in vitro* and in mouse models, to treat melanoma, triple-negative breast cancer, and mesothelioma [19,21,30]. To date, CSPG4 has only been targeted in human trials in the form of mouse anti-idiotypic monoclonal antibodies and vaccines that, though safe, proved mostly ineffective for long-term cancer remission [31-33]. Given the recent success of CAR-based therapies, the development of a safe and effective CAR targeting CSPG4 is certainly appealing.

In this study we have generated CSPG4-targeting CARs from multiple different murine monoclonal antibodies. PBL transduced with these CARs demonstrate cytokine release and cytolytic function when co-cultured with tumor cell lines from different cancer histologies. Of the monoclonal antibodies studied, TP42.1 demonstrated the most consistent expression and reactivity among varying PBL donors. In these experiments, we utilized the well characterized second generation CAR design consisting of the CD28 costimulatory domain linked to the intracellular T cell receptor signaling chain CD3zeta. As CAR technology becomes increasingly sophisticated, the potential benefits various T cell signaling/costimulatory domains, including improved antitumor activity and prolonged survival of modified T cells, will continue to be explored [6,34]. Though it is not yet clear what the optimal combination and arrangement of additional intracellular signaling domains, such as 41BB or CD27 may be, the ability to successfully target tumor cell lines with variety CSPG4-directed CARs is promising [35]. Most recently, Geldres et al., reported data with a different mAb-based CSPG4 CAR, and demonstrated similar biological activity, but did not target CSC [36]. These investigators confirmed the result presented herein that in a large panel of normal tissues (n = 33), CSPG4 expression was not observed by immunohistochemistry. In the data reported herein, we used a more sensitive reverse-phase protein array technology and demonstrated 2 of 4 small bowel samples had signals above background.

CSPG4 expression has previously been demonstrated on CSCs derived from squamous cell carcinoma of the head and neck and basal breast carcinomas [16]. This study demonstrates CSPG4 expression on GSCs as well. GSCs were derived from resected human glioblastomas and have previously been well characterized by proliferation kinetics, immunohistochemistry, histopathology and gene expression profiling [27]. They demonstrated more similarities to human glioblastomas than do traditional tumor cell lines. CSCs have been shown to effect tumor recurrence and development of metastatic disease, exhibit characteristics of self-renewal and resistance to chemotherapy and radiotherapy, and can induce tumor formation in immunodeficient mice [37]. Tumor eradication may ultimately require targeting CSCs, therefore the ability of a therapy to recognize and kill these cells is important. In this study, an anti-CSPG4 CAR demonstrated recognition and cytolysis of multiple GSC lines, a finding with potential therapeutic implications.

CSPG4 could be an attractive immunotherapy target given its expression on a high percentage of melanomas as well as many other cancer histologies and cancer stem cells. Of potential concern though, is the observation of low-level antigen expression on two of 94 normal tissues samples (both from small bowel) as determined by reverse-phase protein array (Figure 3). On-target/off-tumor toxicity is a known property of CAR engineered T cells and has been observed in renal cell carcinoma, colorectal cancer and B cell malignancies [10,38,39]. To limit potential toxicities, it may be possible to target multiple antigens or to engineer T cells with a suicide gene safety switch to potentially manage deleterious recognition of normal tissues.

## Conclusions

Multiple variations of CSPG4-targeting CARs were described in this study, utilizing several murine monoclonal antibodies. CAR-transduced PBL successfully recognized and killed tumor cell lines from multiple different cancer histologies as well as glioblastoma-derived CSCs. CSPG4 has significant potential as an immunotherapy target, as supported by its expression on a high percentage of melanomas and its prevalence in other cancer histologies and CSCs, as well as, its general lack of expression on normal tissues as demonstrated by immunohistochemistry. The recent success of CAR therapies suggests that with appropriate safety modifications, we can be cautiously optimistic regarding the potential development of a CSPG4-specific CAR therapy for the treatment of metastatic cancer.

## Methods

### Tumor and normal tissue samples, cell lines, and lymphocytes

All human cells and tissues were obtained under Internal Review Board approved protocols (National Cancer Institute, Bethesda, MD). Tumor cell lines were grown under standard conditions in Roswell Park Memorial Institute (RPMI) 1640 or Dulbecco’s Modified Eagle Medium (DMEM) (Invitrogen, Carlsbad, CA) with 10% fetal bovine serum (FBS) medium (Sigma Aldrich, St. Louis, MO), 25 mM Hepes (Invitrogen) and 100U/μg per mL of penicillin/streptomycin (Invitrogen) at 37°C in a 5% CO_2_ atmosphere. Peripheral blood lymphocytes (PBL) were collected via leukapharesis. Lymphocytes were maintained in AIM-V medium (Invitrogen) supplemented with 5% human antibody serum (Valley Biomedical, Winchester, VA), 25 mM Hepes (Invitrogen), 100U/μg per mL of penicillin/streptomycin (Invitrogen), and 300 IU/mL interleukin-2 (Aldesleukin, Prometheus, San Diego, CA). Description of tumor cell lines and CSPG4 expression was as previously reported (17–22).

### RNA isolation and RT-PCR

RNA isolation was performed using a RNeasy Mini Kit (Qiagen, Valencia, CA). Reverse transcription (RT) was performed using a High Capacity cDNA Reverse Transcription Kit (Applied Biosystems, Grand Island, NY). Quantitative PCR was performed with the TaqMan Fast Universal PCR Master Mix (Applied Biosystems, Grand Island, NY) and the use of a 7500 Fast Real-Time PCR System (Applied Biosystems, Grand Island, NY). Copy numbers were determined using a standard curve generated from the CSPG4 plasmid and results were normalized against β-actin (ACTB).

### Immunohistochemistry

Immunohistochemistry was performed on a normal tissue panel and tumor slides following paraffinization in xylene and graded alcohol. A panel of anonymous human normal tissue and melanomas obtained from the Tissue Array Research Program (Laboratory of Pathology, National Cancer Institute, Bethesda, MD) was used to construct a “mini-TMA” of 1.5 mm cores. Antigen retrieval was performed in pH 9 AfR buffer (Dako, Carpinteria, CA) for 20 minutes in a pressure cooker. The TP41.2 antibody was used at a concentration of 1:100 at room temperature for 60 minutes, followed by Detection Envision + (Dako) DAB for 10 minutes, followed by dehydration and a coverslip. A negative control with no primary antibody was performed, and a melanoma sample was used as a primary control.

#### Well-based reverse-phase protein array

Total proteins were extracted from three 10 μm frozen tissue sections using T-PER buffer (Pierce Biotechnology) with proteinase inhibitor cocktail (1 tablet/25 ml, Roche). Two μg protein extract from frozen tissue specimen was added to Meso Scale Discovery (MSD, Gaithersburg, MD) Multi-Spot™ plates (MA2400 96 HB Plate), the plate was allowed to dry at room temperature for 90 min, and the plates were subsequently further incubated for 30 min at 37°C. The antigen-coated plates were preincubated with 3% nonfat milk in PBST for 60 min at RT before primary antibody reactions. Anti-CSPG4 (TP41.2) and anti-Actin (Abnova, mouse, clone 3G4-F9) were diluted 1:1000 and 1:5000 respectively, with 3% BSA in PBST, and then incubated overnight at 4°C. After washing with PBST, the plates were incubated for 1 h with goat anti-mouse SULFO-TAG™ antibodies at a dilution of 1:2000 (0.5 μg/ml) with 5% nonfat milk in PBST. The plates were then aspirated and washed three times with PBST. Finally, MSD-T read buffer was added to the plates and they were read on the MSD Sector Imager 2400 reader (Meso Scale Discovery). BSA coated wells were included on each plate as a control for non-specific binding effects. Signal was normalized to actin expression and is expressed as relatively fold over background (water).

### Generation of chimeric antigen constructs, retroviral supernatant production and T-cell transduction

The scFvs derived from the murine mAb 225.28S, TP41.2, 149.53 and G71.1 (references, 23–25, and patent number US 6,924,359) were synthesized as codon-optimized CARs using peptide linkers 218 (sequence G2TSGSGKPGSGEGS) or g4s (sequence GGGGSGGGGSGGGGS) with signaling domains CD28.CD3ζ (Blue Heron Bio, Bothell, WA) [23-25]. The precise antigen binding domains of these mAbs were not determined. The complete scFv sequence was cloned into MSGV-4D5-28z vector backbone after removal of the 4D5 scFv, with production of retroviral supernatant and PBL transduction performed as previously described (the complete amino acid sequence of the CD28-CD3zeta signaling domains used was described in the supplemental data to Zhao et al.) [40].

### Flow cytometry and functional assays

FACS was performed using a conjugated mAb (anti-hNG2/MCSP) specific for human chondroitin sulfate proteoglycan 4 (CSPG4) according to manufacturers’ recommendations (R&D Systems, Minneapolis, MD). To assess for transduction efficiencies, protein L staining was used as previously described [41] as were F(ab’)_2_ fragment goat anti-mouse IgG antibodies (Jackson Immunoresearch, West Grove, PA), according to manufacturers recommendations. Cytokine secretion assays were done as previously described [40]. Cytolysis was assessed by either ^51^Cr assay, as previously described [40], or by CytoTox-Glo™ bioluminescence assay (Promega, Madison, WI), which utilizes the luminogenic AAF-Glo™ Substrate to measure dead-cell protease activity that generates a luminescent signal proportional to the number of lysed cells in a sample. Effector and target cells were coincubated at 37°C for 4 h. Bioluminescence release was measured and specific lysis was calculated according to the following formula: percent specific lysis = [specific release – (spontaneous effector release + spontaneous target release)]/total target release – spontaneous target release × 100%, average of triplicate samples.

### Generation of glioblastoma stem cells

Glioblastoma stem cells (GSCs) were generated from tumors by enzymatic digestion into single cells and subsequent growth in NBE medium, comprised of Neurobasal-A medium (Invitrogen, Carlsbad, CA) supplemented with N2 and B27 (Invitrogen), bFGF and epidermal growth factors (R&D Systems, Minneapolis, MN) as previously described [27,42].

## Abbreviations

ACT: Adoptive cell transfer; CAR: Chimeric antigen receptor; CSC: Cancer stem cell; CSPG4: Chondroitin sulfate proteoglycan 4; DMEM: Dulbecco’s Modified Eagle Medium; FACS: Fluorescence-activated cell sorting analysis; FBS: Fetal bovine serum; GSC: Glioma stem cell; HMW-MAA: High molecular weight-melanoma associated antigen; mAb: Murine monoclonal antibody; PBL: Peripheral blood lymphocytes; scFv: Single-chain variable fragment; RPMI: Roswell Park Memorial Institute; RT: Reverse transcription; RT-PCR: Reverse transcription polymerase chain reaction; TCR: T cell receptor; TIL: Tumor-infiltrating lymphocytes.

## Competing interests

The authors declare that they have no competing interests.

## Authors’ contributions

RB performed the analyses of CSPG4 expression of cells lines and some of the coculture experiments and drafted the manuscript. ZZ performed the cloning, transductions and functional assays of the TP41.2 CAR construct. KL and WB carried out early vector construction and assays. ET performed additional experiments to assess antigen expression in other cell lines. SH performed the immunohistochemical staining of tissue samples. SFR and DAD assisted with cocultures and cytolytic assays. HF oversaw the initial development of the glioblastoma stem cells. SF provided the monoclonal antibodies used in the construction of the CARs. SAR contributed to the design and focus of the study. RM conceived the study and oversaw its design and coordination and assisted with finalization of the manuscript. All authors read and approved the final manuscript.
